# Supplementary material for: MetaRibo-Seq measures translation in microbiomes
Source: Nat Commun. 2020 Jun 29;11:3268. doi: 10.1038/s41467-020-17081-z (PMC7324362; doi:10.1038/s41467-020-17081-z)
Supplement: Supplementary file 10 — Supplementary Data 7 [file 41467_2020_17081_MOESM10_ESM.zip › File2/Confidence_High_Taxonomy/199187_out.krona.html]

Javascript must be enabled to view this page.

members
magnitude
magnitudeUnassigned
count
unassigned
taxon
rank

199187\_out

20

20
2
superkingdom

20
201174
phylum

20
class
1760

order
85004
20

20
31953
family

6
20
1678
genus

SRS012273\_contig\_number\_contig-100\_23231.23231SRS014855\_contig\_number\_contig-100\_33673.33674SRS015578\_contig\_number\_contig-100\_16041.140454SRS019068\_contig\_number\_contig-100\_9793.138806SRS1041137\_contig\_number\_contig-100\_18746.103337SRS971275\_contig\_number\_15308

1680

SRS017521\_contig\_number\_contig-100\_17740.63998SRS018541\_contig\_number\_contig-100\_6496.6497SRS020869\_contig\_number\_7417SRS022071\_contig\_number\_contig-100\_863.171607SRS024331\_contig\_number\_6143SRS050941\_contig\_number\_contig-100\_6936.57752SRS056273\_contig\_number\_26969SRS1041031\_contig\_number\_10937SRS1041033\_contig\_number\_19140SRS1041133\_contig\_number\_18776SRS1055056\_contig\_number\_contig-100\_15626.54599SRS892882\_contig\_number\_contig-100\_19069.19070SRS893366\_contig\_number\_contig-100\_5943.39497
species
13

species
1686
1

subspecies

SRS074670\_contig\_number\_contig-100\_4847.39226
630129
1
